# Association of Preeclampsia With Incident Stroke in Later Life Among Women in the Framingham Heart Study

**DOI:** 10.1001/jamanetworkopen.2021.5077

**Published:** 2021-04-26

**Authors:** Adam de Havenon, Alen Delic, Eric Stulberg, Nazanin Sheibani, Greg Stoddard, Heidi Hanson, Lauren Theilen

**Affiliations:** 1 Department of Neurology, University of Utah, Salt Lake City; 2 Department of Epidemiology, University of Utah, Salt Lake City; 3 Department of Surgery, University of Utah, Salt Lake City; 4 Department of Obstetrics and Gynecology, University of Utah, Salt Lake City

## Abstract

**Importance:**

Contemporary research suggests an association between preeclampsia and later-life stroke among women. To our knowledge, no research to date has accounted for the time-varying nature of shared risk factors for preeclampsia and later-life stroke incidence.

**Objective:**

To assess the relative risk of incident stroke in later life among women with and without a history of preeclampsia after accounting for time-varying covariates.

**Design, Setting, and Participants:**

This population-based cohort study was a secondary analysis of data from the Framingham Heart Study, which was conducted from 1948 to 2016. Women were included in the analysis if they were stroke free at enrollment and had a minimum of 3 study visits and 1 pregnancy before menopause, hysterectomy, or age 45 years. Data on vascular risk factors, history of preeclampsia, and stroke incidence were collected biannually. Participants were followed up until incident stroke or censorship from the study. Marginal structural models were used to evaluate the relative risk of incident stroke among participants with and without a history of preeclampsia after accounting for time-varying covariates. Data were analyzed from May 2019 to December 2020.

**Exposures:**

Presence or absence of preeclampsia among women with 1 or more pregnancies.

**Main Outcomes and Measures:**

Incident stroke in later life.

**Results:**

A total of 1435 women (mean [SD] age, 44.4 [7.7] years at the beginning of the study; 100% White) with 41 422 person-years of follow-up were included in the analytic sample. Of those, 169 women had a history of preeclampsia, and 231 women experienced strokes during follow-up. At baseline, women with preeclampsia were more likely to be younger, to be receiving cholesterol-lowering medications, to have lower cholesterol and higher diastolic blood pressure, and to currently smoke. The association between preeclampsia and stroke in the marginal structural model was only evident when adjustment was made for all vascular risk factors over the life course, which indicated that women with a history of preeclampsia had a higher risk of stroke in later life compared with women without a history of preeclampsia (relative risk, 3.79; 95% CI, 1.24-11.60).

**Conclusions and Relevance:**

The findings of this cohort study suggest that preeclampsia may be a risk factor for later-life stroke among women after adjustment for time-varying vascular and demographic factors. Future research is warranted to fully explore the mediation of this association by midlife vascular risk factors.

## Key Points

**Question:**

Is preeclampsia associated with later-life stroke among women after accounting for time-varying covariates?

**Findings:**

In this cohort study of 1435 women with and without a history of preeclampsia who participated in the Framingham Heart Study, after adjustment for age and time-varying vascular risk factors, women with a history of preeclampsia had a more than 3-fold higher risk of experiencing a stroke in later life compared with women who did not have a history of preeclampsia.

**Meaning:**

The findings suggest that preeclampsia may be an independent risk factor for later-life stroke among women after controlling for vascular risk factors across the life course.

## Teaser

This cohort study uses data from the Framingham Heart Study and an analytic model that accounts for time-varying factors to assess the risk of stroke in later life among women with and without a history of preeclampsia in pregnancy.

## Introduction

Hypertensive disorders of pregnancy are a major cause of morbidity and mortality in the peripartum period and predispose women to an elevated risk of cardiovascular, cerebrovascular, and renal disease later in life.^1^ Preeclampsia, broadly defined as new-onset hypertension during pregnancy with end-organ damage, is a common hypertensive disorder of pregnancy occurring in up to 8% of pregnancies. Preeclampsia can result in acute cerebrovascular complications, including stroke and intracranial vasculopathy,^2^ and has been associated with an increase in the risk of stroke in later life. However, existing research has not fully accounted for time-varying midlife risk factors that could bias the association between preeclampsia and later-life stroke.^2,3,4,5^ To address this limitation, we used data from the Framingham Heart Study (FHS), which enrolled 2873 women who had up to 32 follow-up visits every other year. At each visit, data were collected on cardiovascular risk factors and stroke incidence.^6^ We hypothesized that preeclampsia was associated with later-life stroke after adjustment for time-dependent covariates at every study visit.

## Methods

### Cohort

This cohort study was a secondary analysis of the FHS cohort; the data set was obtained in December 2018 from the Biologic Specimen and Data Repository Information Coordinating Center of the National Heart, Lung, and Blood Institute.^7^ The FHS was an epidemiological study of cardiovascular disease conducted from 1948 to 2016. White participants aged 28 to 74 years were followed up from baseline until death, loss to follow-up, or the last study visit in 2016.^8^ The present study was approved by the institutional review board of the University of Utah, with a waiver of informed consent because of the use of deidentified publicly available data. This study followed the Strengthening the Reporting of Observational Studies in Epidemiology (STROBE) reporting guideline for cohort studies.

The study exposure was the presence or absence of preeclampsia during 1 or more pregnancies before enrollment in the FHS. Preeclampsia was considered present if participants responded affirmatively to a yes or no question at study enrollment regarding their history of toxemia, the prevailing nomenclature for preeclampsia at the time.^9^ We included women who were stroke free at study entry and had a minimum of 3 study visits and 1 pregnancy before menopause, hysterectomy, or age 45 years, which was a reliable cutoff age for the loss of fertility in that era.^10,11^ To limit bias, we excluded 8 women who had a pregnancy that was not complicated by preeclampsia at baseline but who subsequently reported preeclampsia during the outcome period.

### Outcomes and Covariates

The study’s outcome was incident nonfatal or fatal stroke, which was further stratified by ischemic and hemorrhagic stroke. Stroke occurrence was centrally adjudicated in the FHS. Participants were followed up at biannual visits, and data on cardiovascular covariates associated with the risk of stroke were collected^12^; these covariates included blood pressure, blood glucose level, lipid levels, current smoking status (yes or no), weight (converted to kilograms from pounds, which was the original unit of measurement reported in the FHS), and age. With regard to lipids, total cholesterol and phospholipid levels were recorded and included in our analysis because both factors were reported in the FHS to be associated with cardiovascular events, including stroke.^13,14^

We arranged the study data such that, across 65 years of potential follow-up, participants were observed in continual 2-year intervals until they either experienced a stroke event (n = 231) or were censored from the study (n = 1435). As the participants were followed up through biennial visits, their exposure and covariate histories were continuously updated at each visit. Missed visits were addressed by carrying the last known value forward, which resulted in imputation of less than 5% of missing values.

### Statistical Analysis

Confounding was introduced because covariates, such as the development of hypertension, changed with time. The theoretical framework of the time-varying covariates observed in the analysis is shown in the Figure. Although we do not know the exact associations between preeclampsia and the covariates, the Figure illustrates their potential complexity. Blood pressure can be used as an example of a time-varying covariate. Proper control for blood pressure as a confounder would include controlling for the confounding of blood pressure on the exposure-outcome association at visit 1 as well as the exposure status at visit 1 associated with the level of confounding blood pressure at visit 2. Improper control of blood pressure at visit 2 would induce bias, as this measurement is an important factor in the association between the exposure and outcome.

**Figure.**
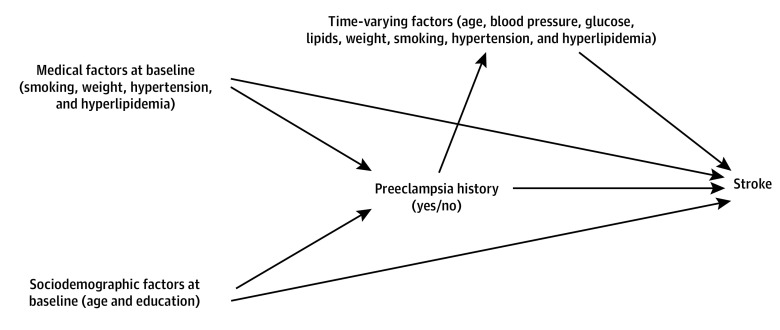
Directed Acyclic Graph of Assumptions Regarding the Association Between Preeclampsia, Risk Factors, and Stroke

Standard survival analysis methods may not have optimally controlled for time-varying covariates.^15,16,17^ In addition, because the system of covariates was complex, with possible interactions over decades of follow-up, traditional regression analysis would have produced a biased estimate because of the difficulty of model specification.^18,19^ Therefore, we used marginal structural models (MSMs) to account for time-varying covariates and censoring, with a fixed exposure plan for preeclampsia.^20,21^

Inverse probability of treatment weighting is the most common method used to address time-varying covariates in MSMs. In this method, exposure (ie, treatment) and censoring weights are calculated for each participant to account for uneven distributions of covariates across exposure groups. Exposure weights represent the probability of receiving exposure given a participant’s distinct covariate history.^18^ Censoring weights account for possible attrition in censoring between the exposure groups. A pseudopopulation is created by multiplying the 2 sets of calculated weights. This results in a pseudopopulation that has balance in these characteristics among both exposure groups.^22^ We set the truncation by default at the 0.5 and 99.5 percentiles to not bias the truncation and estimates of the final models.^23,24^ The range of the nontruncated weights for the preeclamptic and nonpreeclamptic groups were 0.12 × 10^10^ and 0.04 × 10^17^, respectively. After truncation, the range of weights for the preeclamptic and nonpreeclamptic groups were 0.25 to 195.06 and 0.29 to 221.38, respectively. Marginal structural models use these weights to reflect overrepresentation or underrepresentation of participants with certain characteristics compared with a target population.

The MSM is then applied to the pseudopopulation via a pooled logistic regression model, in which the outcome of interest can be estimated using the calculated weights. This regression model uses the balanced pseudopopulation to generate a hypothetical scenario of events that may occur if a certain participant has a history of preeclampsia vs events that may occur if the same participant has never had preeclampsia. The final result is an estimate of the hazard ratio, which can equivalently be interpreted as the relative risk (RR) of stroke for those with a history of preeclampsia relative to those without a history of preeclampsia.^25^ We also examined the association between preeclampsia and stroke in logistic regression models adjusted for baseline covariates to compare those data with results from the MSM model. Stata version 16.1 (StataCorp LLC) was used for data management and analysis. The level of significance was defined as *P* = .05, and all hypothesis tests were 2-sided. Data were analyzed from May 2019 to December 2020.

## Results

The analysis included 1435 women (mean [SD] age, 44.4 [7.7] years; 100% White) enrolled in the FHS who had an accumulated 51 600 person-years of observation. A history of preeclampsia was present in 169 women (11.8%), and the outcome of stroke occurred in 231 women (16.1%) at a mean (SD) of 32.4 (1.1) years from baseline. Stroke events occurred in 30 of 169 women (17.8%) with a history of preeclampsia and in 201 of 1266 women (15.9%) without a history of preeclampsia. At baseline, women with a history of preeclampsia vs those without a history of preeclampsia were substantially younger (mean [SD] age, 44.4 [7.7] years vs 48.9 [7.7] years, respectively) and more likely to be receiving treatment for an elevated cholesterol level (4 of 169 women [2.4%] vs 5 of 1266 women [0.4%]), to currently smoke (81 of 169 women [47.9%] vs 465 of 1266 women [36.7%]), and to have lower serum total cholesterol levels (mean [SD], 226.2 [48.3] mg/dL vs 234.4 [44.8] mg/dL) and higher diastolic blood pressure (86.4 [13.3] mm Hg vs 83.6 [12.4] mm Hg). At the final visit, women with a history of preeclampsia had substantially higher blood glucose levels (mean [SD], 113.9 [55.2] mg/dL vs 104.4 [45.7] mg/dL) and lower phospholipid levels (mean [SD], 282.6 [50.4] mg/dL vs 291.8 [48.8] mg/dL) and were more likely to be receiving treatment for hypertension (96 of 169 women [56.8%] vs 565 of 1266 women [44.6%]) and for an elevated cholesterol level (19 of 169 women [11.2%] vs 36 of 1266 women [2.8%]) compared with women without a history of preeclampsia (Table 1). (To convert total cholesterol levels to millimoles per liter, multiply by 0.0259; to convert glucose levels to millimoles per liter, multiply by 0.0555.)

**Table 1.** Characteristics of Women With and Without Preeclampsia at Baseline and Final Visits

Characteristic^**a**^	Mean (SD)					
	Baseline visit			Final visit		
	With preeclampsia (n = 169)	Without preeclampsia (n = 1266)	*P* value	With preeclampsia (n = 169)	Without preeclampsia (n = 1266)	*P* value
Age, y	44.4 (7.7)	48.9 (7.7)	<.001	77.5 (11.0)	77.5 (10.2)	.95
Weight, kg^b^	65.2 (12.4)	65.1 (11.3)	.94	62.3 (14.9)	61.2 (12.4)	.31
Total cholesterol, mg/dL	226.2 (48.3)	234.4 (44.8)	.03	256.5 (56.5)	263.6 (52.7)	.10
Blood glucose, mg/dL	81.3 (12.9)	83.0 (21.0)	.30	113.9 (55.2)	104.4 (45.7)	.01
Phospholipids, mg/dL	200.6 (53.4)	205.2 (55.9)	.31	282.6 (50.4)	291.8 (48.8)	.02
Blood pressure, mm Hg						
Systolic	138.3 (26.1)	134.9 (24.2)	.09	147.5 (25.9)	145.3 (25.3)	.28
Diastolic	86.4 (13.3)	83.6 (12.4)	.006	74.4 (14.0)	74.9 (13.3)	.64
Receiving treatment for hypertension, No. (%)	13 (7.7)	62 (4.9)	.13	96 (56.8)	565 (44.6)	.003
Receiving treatment for hyperlipidemia, No. (%)	4 (2.4)	5 (0.4)	.002	19 (11.2)	36 (2.8)	<.001
Current smoking, No. (%)	81 (47.9)	465 (36.7)	.005	28 (16.6)	218 (17.2)	.84

^a^ SI conversion factors: To convert total cholesterol to millimoles per liter, multiply by 0.0259; glucose to millimoles per liter, multiply by 0.0555.

^b^ Weight has been converted to kilograms from pounds, which was the original unit of measurement reported in the Framingham Heart Study. To convert from kilograms to pounds, multiply by 2.205.

Table 2 depicts the association between preeclampsia and stroke using different models. In the unadjusted logistic regression model, preeclampsia did not have a statistically significant association with later-life stroke (odds ratio [OR], 1.14; 95% CI, 0.75-1.74; *P* = .53). The association remained null in the logistic model after covariate adjustment (OR, 1.27; 95% CI, 0.80-2.00; *P* = .31), while in the MSM model, those with preeclampsia had a relative risk of 3.79 (95% CI, 1.24-11.60; *P* = .02) for the risk of experiencing later-life stroke compared with those without preeclampsia.

**Table 2.** Association Between Preeclampsia and Risk of Stroke Among Women in the Framingham Heart Study

Variable	Participants, No. (%) (N = 1435)	Person-years	Logistic model unadjusted		Logistic model adjusted for baseline factors^a^		MSM model adjusted for time-varying factors from baseline to final visit^b^	
			OR (95% CI)	*P* value	OR (95% CI)	*P* value	RR (95% CI)	*P* value
History of preeclampsia								
No	1266 (88.2)	36 076	1 [Reference]	.53	1 [Reference]	.31	1 [Reference]	.02
Yes	169 (11.8)	5346	1.14 (0.75-1.74)		1.27 (0.80-2.00)		3.79 (1.24-11.60)	

Abbreviations: MSM, marginal structural model; OR, odds ratio; RR, relative risk.

^a^ Adjusted for educational level (less than high school, high school graduate with some college, other post–high school educational degree [eg, business school, nursing school, and music or art school], and college graduate or higher) and baseline characteristics at enrollment in the Framingham Heart Study, including age (continuous), weight (continuous), total cholesterol level (continuous), blood glucose level (continuous), systolic blood pressure (continuous), diastolic blood pressure (continuous), receipt of treatment for hypertension (yes or no), receipt of treatment for hyperlipidemia (yes or no), and current smoking status (yes or no).

^b^ Adjusted for educational level, baseline characteristics, and potential mediating factors, including age (continuous) and weight (continuous) at follow-up visit, total cholesterol level (continuous), blood glucose level (continuous), systolic blood pressure (continuous), diastolic blood pressure (continuous), receipt of treatment for hypertension (yes or no), receipt of treatment for hyperlipidemia (yes or no), and current smoking status (yes or no).

After stratification by stroke subtype, the final model indicated an association between preeclampsia and ischemic stroke (RR, 4.13; 95% CI, 1.11-15.40; *P* = .03) (Table 3) but no association between preeclampsia and hemorrhagic stroke (RR, 4.12; 95% CI, 0.93-18.30; *P* = .06). Age, weight, and blood pressure were important confounders (Table 4). Full covariate adjustment without age produced a null association (RR, 0.79; 95% CI, 0.51-1.23; *P* = .30) as did adjustment for age alone (RR, 2.62; 95% CI, 0.47-14.46; *P* = .27). Full covariate adjustment excluding only weight also yielded a null association (RR, 2.28; 95% CI, 0.85-6.11; *P* = .10), as did full covariate adjustment excluding only blood pressure (RR, 2.97; 95% CI, 0.93-9.52; *P* = .07).

**Table 3.** Final Estimate of the Association Between Preeclampsia and Stroke by Stroke Type

Stroke type	Events, No.	Relative risk (95% CI)^a^	*P* value
All	231	3.79 (1.24-11.60)	.02
Ischemic	201	4.13 (1.11-15.40)	.03
Hemorrhagic	30	4.12 (0.93-18.30)	.06

^a^ Adjusted for educational level, baseline characteristics, and potential mediating factors, including age (continuous) and weight (continuous) at follow-up visit, total cholesterol level (continuous), blood glucose level (continuous), systolic blood pressure (continuous), diastolic blood pressure (continuous), receipt of treatment for hypertension (yes or no), receipt of treatment for hyperlipidemia (yes or no), and current smoking status (yes or no).

**Table 4.** Association Between Preeclampsia and Stroke in the Marginal Structural Model

Model and covariates	Relative risk (95% CI)	*P* value
Null with age	2.62 (0.47-14.46)	.27
Full model without age	0.79 (0.51-1.23)	.30
Full model without weight	2.28 (0.85-6.11)	.10
Full model without cholesterol	3.60 (1.18-10.99)	.02
Full model without blood glucose	3.16 (1.03-9.70)	.04
Full model without blood pressure	2.97 (0.93-9.52)	.07
Full model with all covariates^a^	3.79 (1.24-11.60)	.02

^a^ Adjusted for educational level, baseline characteristics, and potential mediating factors, including age (continuous) and weight (continuous) at follow-up visit, total cholesterol level (continuous), blood glucose level (continuous), systolic blood pressure (continuous), diastolic blood pressure (continuous), receipt of treatment for hypertension (yes or no), receipt of treatment for hyperlipidemia (yes or no), and current smoking status (yes or no).

## Discussion

After adjustment for time-varying confounders and censoring, we found that preeclampsia among White women was associated with significant increases in the long-term risk of incident stroke. As with all observational studies, causality could not be established because of unmeasured confounding, and the results of this study are not generalizable to non-White racial or ethnic groups. However, we were able to use the longitudinal granularity of the FHS data to provide insights into the later-life risk that having a preeclamptic pregnancy may confer. Although the logistic regression models without time-varying covariates did not indicate a significant association, they fail to capture the enduring changes that occur after preeclampsia.^1^ Based on the assumptions of the MSM framework, the estimates of our fitted MSM are interpretable as the association that would have been observed in a clinical trial examining the association of preeclampsia with stroke among a randomized cohort of women with at least 1 pregnancy. Such a clinical trial would be unethical to perform; therefore, we used the MSM framework to create a pseudopopulation in which the possible confounder distributions were balanced across exposure groups to estimate an unbiased association between preeclampsia and subsequent stroke risk.

Biomarkers of impaired vascular health, including increased cerebral small vessel disease and carotid intima-media thickness, have been observed years to decades after preeclampsia.^2^ However, the time-dependent confounding by each patient’s vascular health during the life course presents distinct methodological challenges that published studies of preeclampsia and later-life stroke have not addressed.^3,4,5^ Our results are consistent with previous research on preeclampsia that excluded strokes occurring during pregnancy or puerperium. The Stroke Prevention in Young Women study reported that a history of preeclampsia had an OR of 1.63 for ischemic stroke among a sample of 682 women; however, the study lacked data on vascular risk factors during midlife.^26^ The Million Women Study reported that women with a history of a hypertensive disorder of pregnancy had a relative risk of 1.18 to 1.86 for ischemic stroke, but the study lacked longitudinal data.^4^ Additional studies using administrative data sets or registries have found an association between preeclampsia and later-life stroke, but we are unaware of any studies that were able to account for biannual risk factor profiles throughout midlife.^2,27^

The most substantial confounder in our analysis was participant age, with weight and blood pressure also being notable confounders. However, adjusting for age alone was insufficient to establish an association between preeclampsia and stroke. Establishing this association requires additional adjustment for vascular risk factors, including blood pressure, cholesterol level, blood glucose level, smoking status, and weight. Thus, it is the interplay of increasing age and the accumulation of vascular risk factors that likely creates the association between preeclampsia and stroke. Although age cannot be altered, our study results suggest that improved midlife control of hypertension, hyperlipidemia, hyperglycemia, and other vascular risk factors has the potential to mitigate the later-life risk of stroke among women with a history of preeclampsia.

We cannot provide any definite conclusions regarding the mechanism of increased risk of stroke among women with a history of preeclampsia. Based on our analysis using the MSM model specification, there may be a complex interplay between preeclampsia and the accumulation of comorbidities throughout one’s life that factors into the risk of subsequent stroke. Formal longitudinal causal mediation analysis could be used to examine the possible mechanisms underlying the progression from exposure to outcome that may be present in this study but are beyond the scope of the current analysis. This study instead illustrates the ways in which MSMs can be properly used to estimate the association between an exposure and an outcome (ie, preeclampsia and stroke) when there is high likelihood of bias induced by time-varying covariates.

### Limitations

This study has several limitations. The main limitation is that the history of preeclampsia was patient reported and could be susceptible to ascertainment bias. Self-reported history of preeclampsia has been reported to be highly specific but not sensitive^28,29^; thus, we would expect this inaccuracy to bias our findings toward the null. Because the exposure was recorded as a binary variable at study entry, we do not know the dates of preeclampsia exposure, although the relatively young age of participants at enrollment meant that the stroke events occurred long after the exposure. We also did not explore the association between stroke and the number of preeclamptic pregnancies or recurrent preeclampsia during the study period, which could have provided insight regarding a dose effect but would have required a larger cohort.

The original FHS cohort only enrolled White participants, which limits the generalizability of our findings. We did not have detailed or consistent data on additional vascular risk factors, most importantly physical activity and diet. Despite these limitations, the strengths of our study are notable, primarily the extensive data on vascular risk factor control through midlife, which is a distinct attribute when evaluating this association. We also used a statistical model that was capable of analyzing the granular data in the FHS.

## Conclusions

In this cohort study, White women with a history of preeclampsia had more than 3 times the risk of later-life stroke compared with those without a history of preeclampsia. Because the FHS enrolled only White participants, these results are not generalizable to other racial or ethnic groups. The stroke events occurred at a mean of more than 3 decades after the exposure, suggesting that aggressive medical management of vascular risk factors during midlife has the potential to reduce the risk of stroke. Research is needed to explore the practical implications of this association, particularly regarding the implementation of additional monitoring of vascular health among women with a history of preeclampsia and the use of lower thresholds for medical and lifestyle interventions to improve vascular health.
